# Correction: Contribution of IL-17–producing γδ T cells to the efficacy of anticancer chemotherapy

**DOI:** 10.1084/jem.2010026902032026c

**Published:** 2026-02-12

**Authors:** Yuting Ma, Laetitia Aymeric, Clara Locher, Stephen R. Mattarollo, Nicolas F. Delahaye, Pablo Pereira, Laurent Boucontet, Lionel Apetoh, François Ghiringhelli, Noëlia Casares, Juan José Lasarte, Goro Matsuzaki, Koichi Ikuta, Bernard Ryffel, Kamel Benlagha, Antoine Tesnière, Nicolas Ibrahim, Julie Déchanet-Merville, Nathalie Chaput, Mark J. Smyth, Guido Kroemer, Laurence Zitvogel

Vol. 208, No. 3 | https://doi.org/10.1084/jem.20100269 | March 7, 2011

The authors regret that the upper right and lower right cytofluorometric dot plots in Fig. S2 F were inadvertently duplicated in their original article, likely due to an error during figure assembly. The authors have reanalyzed the original raw data with FlowJo version 10.3 to replace all graphs in Fig. S2 F. The original and corrected Fig. S2 are shown here, and the sentence “The original FACS file was reanalyzed with FlowJo v10.3” has been added to the end of the legend. This error does not affect the findings of the manuscript. The supplemental material PDF has been replaced online, and the error remains only in PDFs downloaded before February 3, 2026.

**Figure:**
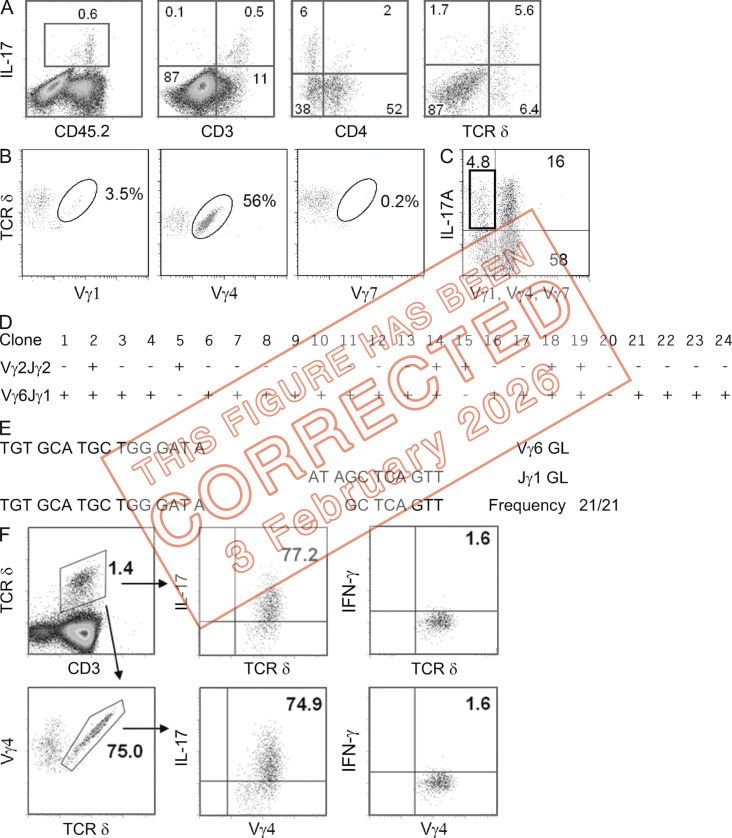


**Figure S2.**
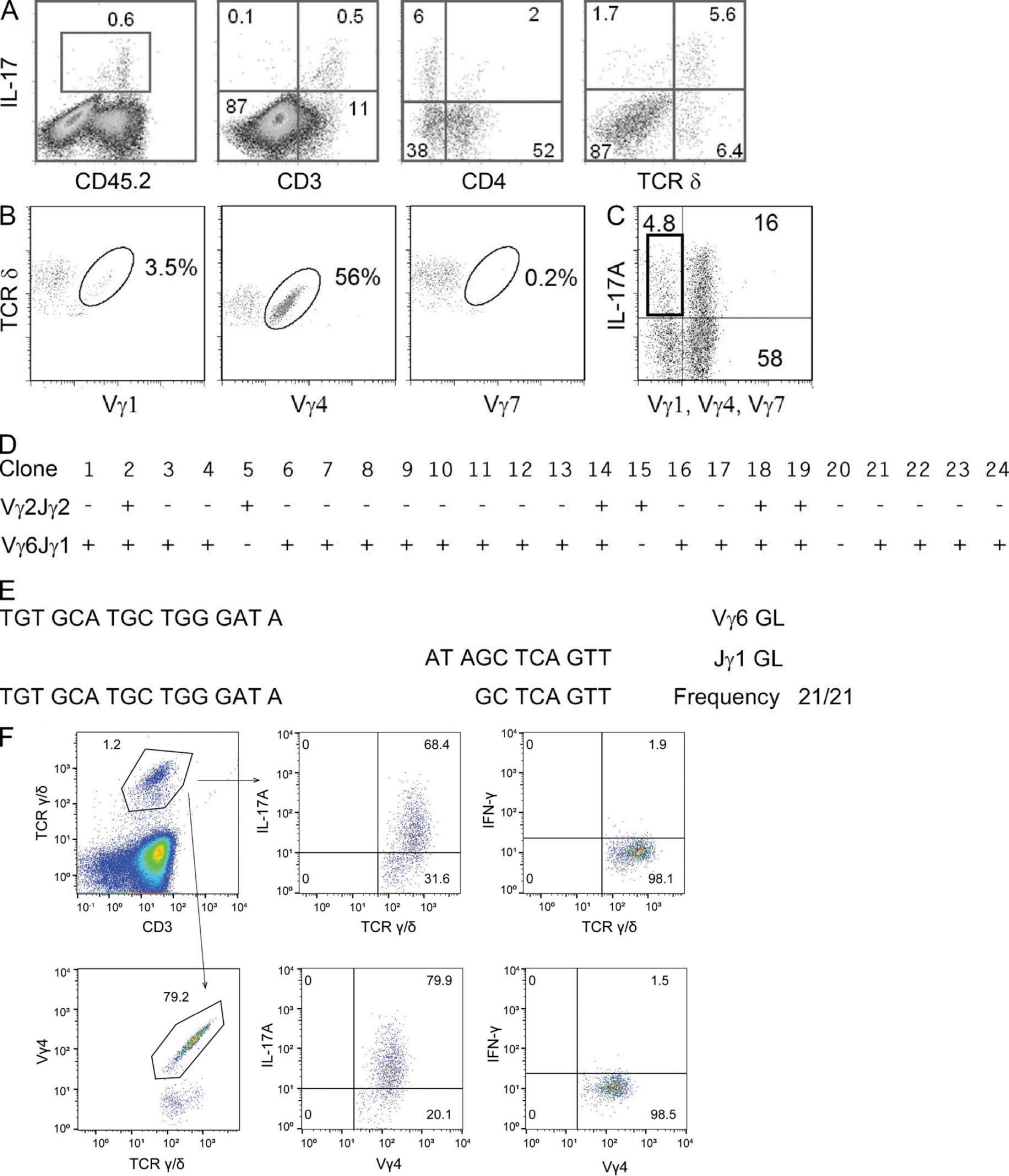
**Vγ chain usage by γδ T17 cells in tumor bed and skin draining LNs of naive mice. (A)** CD45, CD3, CD4, and TCR δ expression by IL-17A–producing cells from tumor beds of mice 8 d after chemotherapy. **(B)** Vγ usage in live CD45^+^ CD3^+^ TCR δ^+^ IL-17^+^ cells in tumor beds after DX. Numbers represent the percentage of Vγ1^+^, Vγ4^+^, or Vγ7^+^ cells among TCR δ^+^ IL-17^+^ cells as indicated. One experiment representative of three is shown. **(C–E)** Individual Vγ1^−^Vγ4^−^Vγ7^−^ γδ T17 TILs as gated in (C) were sorted in PCR plates, and DNA was amplified with primers specific for Vγ2-Jγ2 or Vγ6-Jγ1 rearrangements. **(D)** Presence (+) or absence (−) of specific amplification bands in 24 clones analyzed. **(E)** Junctional sequences of the Vγ6-Jγ1 amplifications present in 21 clones in D. GL denotes the germline sequences of the Vγ6 and Jγ1 ends as indicated. **(F)** LN cells from naive mice were stimulated with PMA/ionomycin, and total γδ T cells (top) or Vγ4^+^ cells (bottom) were gated and analyzed for intracellular IL-17 (middle) or IFN-γ (right) by FACS. Numbers indicate the percentage of cells inside the gates. One experiment representative of four is shown. **The original FACS file was reanalyzed with FlowJo v10.3.**

